# SARS-CoV2 infection impairs the metabolism and redox function of cellular glutathione

**DOI:** 10.1016/j.redox.2021.102041

**Published:** 2021-06-10

**Authors:** Desirée Bartolini, Anna Maria Stabile, Sabrina Bastianelli, Daniela Giustarini, Sara Pierucci, Chiara Busti, Carmine Vacca, Anna Gidari, Daniela Francisci, Roberto Castronari, Antonella Mencacci, Manlio Di Cristina, Riccardo Focaia, Samuele Sabbatini, Mario Rende, Antimo Gioiello, Gabriele Cruciani, Ranieri Rossi, Francesco Galli

**Affiliations:** a Department of Pharmaceutical Sciences, University of Perugia, Nutrition and Clinical Biochemistry Lab, Via Del Giochetto, Monteluce, Perugia, Italy; b Department of Medicine and Surgery, Section of Human, Clinical and Forensic Anatomy, School of Medicine, University of Perugia, P.le Lucio Severi, 1, Sant’Andrea Delle Fratte, 06132, Perugia, Italy; c Department of Medicine and Surgery, Clinic of Infectious Diseases, University of Perugia, Perugia, Italy; d Department of Biotechnology, Chemistry and Pharmacy, University of Siena, Via A. Moro 2, I-53100, Siena, Italy; e Department of Chemistry, Biology and Biotechnology, University of Perugia, Via Elce di Sotto 8, 06123, Perugia, Italy; f Department of Medicine and Surgery, Microbiology Unit, University of Perugia, 06123, Perugia, Italy; g Department of Medicine and Surgery, Medical Microbiology Section, University of Perugia, 06129, Perugia, Italy

**Keywords:** COVID-19, SARS-CoV-2, Glutathione, Thiols, Nrf2, Protein glutathionylation

## Abstract

Viral infections sustain their replication cycle promoting a pro-oxidant environment in the host cell. In this context, specific alterations of the levels and homeostatic function of the tripeptide glutathione have been reported to play a causal role in the pro-oxidant and cytopathic effects (CPE) of the virus. In this study, these aspects were investigated for the first time in SARS-CoV2-infected Vero E6 cells, a reliable and well-characterized in vitro model of this infection. SARS-CoV2 markedly decreased the levels of cellular thiols, essentially lowering the reduced form of glutathione (GSH). Such an important defect occurred early in the CPE process (in the first 24 hpi). Thiol analysis in N-acetyl-Cys (NAC)-treated cells and membrane transporter expression data demonstrated that both a lowered uptake of the GSH biosynthesis precursor Cys and an increased efflux of cellular thiols, could play a role in this context. Increased levels of oxidized glutathione (GSSG) and protein glutathionylation were also observed along with upregulation of the ER stress marker PERK. The antiviral drugs Remdesivir (Rem) and Nelfinavir (Nel) influenced these changes at different levels, essentially confirming the importance or blocking viral replication to prevent GSH depletion in the host cell. Accordingly, Nel, the most potent antiviral in our in vitro study, produced a timely activation of Nrf2 transcription factor and a GSH enhancing response that synergized with NAC to restore GSH levels in the infected cells. Despite poor in vitro antiviral potency and GSH enhancing function, Rem treatment was found to prevent the SARS-CoV2-induced glutathionylation of cellular proteins. In conclusion, SARS-CoV2 infection impairs the metabolism of cellular glutathione. NAC and the antiviral Nel can prevent such defect in vitro.

## Graphical abstract

Schematic representation of the alterations that SARS-CoV2 infection generates in the metabolism and redox function of cellular glutathione.

**Figure:**
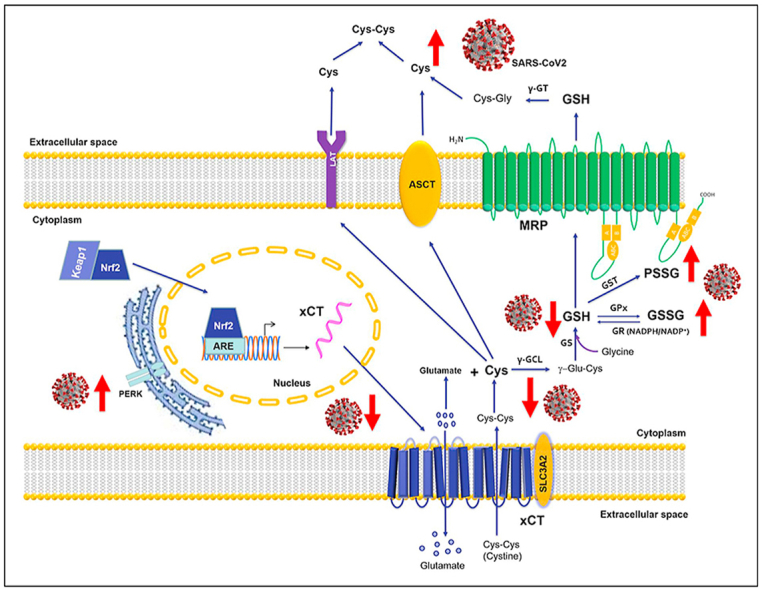

## 1 Introduction

Severe Acute Respiratory Syndrome Coronavirus 2 (SARS-CoV2) is responsible for the Coronavirus disease 2019 (COVID-19) [1], which can present in a significant number of cases, and especially in the elderly and comorbid patients, as a severe respiratory distress syndrome with high risk of disabilities and even death [2,3].

Although the pathophysiology of SARS-CoV2 seems to differ in several ways from that of SARS-CoV and other human coronaviruses with seasonal diffusion, their capability to induce cellular damage and a cytopathic effect (CPE), follows a common mechanism [4,5]. More in detail, the replicative process of many viruses, including the members of family Coronaviridae, is reported to stimulate the unfolded protein response (UPR) and endoplasmic reticulum (ER) stress, with consequent activation of the apoptotic death program of the host cell (reviewed in Refs. [[6], [7], [8]]). All these cellular responses to viral infections and others that are alternative to cell death and disposal, such as autophagy and mitophagy, are highly regulated processes under the influence of the cellular redox (reviewed elsewhere in Refs. [[9], [10], [11]]).

The tripeptide glutathione and its enzymatic system of detoxification and redox homeostasis of protein cysteine (Cys) residues, have a prominent position in the pecking order of cellular systems that control redox-dependent processes, including ER stress and apoptosis [12,13].

Different types of viruses, including influenza, HIV and Coronaviruses, have been described to actively promote a pro-oxidant environment in the infected cell, actively interfering with its redox homeostasis and antioxidant defense systems (reviewed in Ref. [14]). Studies in the influenza and Sendai virus suggest that the depletion of the reduced form of the cellular glutathione (GSH) plays a main role in promoting these alterations, representing an early event in the infection process important in sustaining viral replication and assembly [15,16]. Moreover, for some viral infections, such as HIV, influenza and HSV, cellular GSH depletion represents a mechanism for the virus to evade the immune response (reviewed in Refs. [14,17,18]). Again, the infection process of the influenza virus and its pro-oxidant effects in the host cell, are both reduced by the treatment with GSH analogues in *in vitro* models and *in vivo* in old mice and in lethally-infected mice [15,19], and promising results on these agents have also been reported in other experimental models of viral infection [17]; again, treatments with Cys analogues, such as N-acetyl cysteine (NAC), have successfully been adopted to replenish blood GSH and to improve immune and metabolic functions in HIV-infected patients [20], and NAC is now under clinical investigation in COVID-19, holding potential to prevent severe complications especially in the elderly and comorbid patients (recently reviewed in Ref. [21]).

Mechanistic aspects of GSH depletion in the infected cell remain elusive. These may include a leakage of this tripeptide during the exocytosis process of virus-carrying vesicles, and a preferential incorporation of cellular Cys into the viral proteins that later in the viral cycle could contribute to decrease the biosynthesis of GSH and consequently the redox homeostasis of protein Cys residues [16]. Under these circumstances, the formation of mixed disulfides on the cellular proteins (mainly occurring by glutathionylation), can sustain CPE, promoting abnormal protein damage and ER stress, as well as uncontrolled signal transduction throughout cell cycle regulation and death pathways of the host cell (reviewed in Ref. [22]).

These premises may lead to hypothesize that SARS-CoV2 infection interferes with the levels and metabolism of GSH in the host cell, as well as with the role that this tripeptide plays in the homeostatic control of the cellular redox and extracellular thiols (more details on this hypothesis have recently been presented in Ref. [21]); the investigation of these biochemical aspects during the infectious cycle and CPE of this Coronavirus, may lead to identify novel therapeutic targets and measures of prevention of COVID-19. Accordingly, this hypothesis was explored in the present in vitro study on SARS-CoV2 infected VERO E6 cells during the stimulation of cellular thiol metabolism with NAC and the treatment with different antiviral agents that have already been investigated for an application in COVID-19 therapy, including Remdesivir (Rem), Nelfinavir (Nel) and other viral protease inhibitors.

## 2 Material and methods

### 2.1 Virus isolation

All the experiments with SARS-CoV2 were performed in Biological Safety Level 3 (BSL-3) virology laboratory of “Santa Maria della Misericordia” Hospital, Perugia, Italy. The SARS-CoV-2 strain was isolated as previously described [23], utilizing a nasopharyngeal swab from a symptomatic patient that was collected according with the declaration of Helsinki. The study was approved by the local ethics committee. Briefly, 150 μL of universal transport medium (UTM) of the nasopharyngeal swab were incubated for 1 h at room temperature with a solution (1:1 vol/vol) containing 1% penicillin +1% streptomycin to remove bacterial/fungal contaminations. The suspension was inoculated in kidney epithelial cells extracted from an African green monkey (Vero-E6) cells and then incubated at 37 °C for 2 h. At the end of the incubation period, the inoculum was removed and a media with penicillin 1%, streptomycin 1% plus FBS 1% was added. After 3 days, a clear CPE was detected, the supernatant was harvested, and SARS-CoV2 identification was performed by RT-qPCR. Viral titer was determined by Median Tissue Culture Infectious Dose (TCID50) endpoint dilution and stock aliquots were stored at −80 °C. The stock virus titer was 3.16 × 10^7^ TCID50/mL and the frozen aliquots were thawed immediately before each experiment.

### 2.2 Cell culture conditions and treatments

Vero-E6 cells were grown in minimal essential medium (MEM, Invitrogen, Life Technologies) supplemented with 10% FBS (Gibco, Thermo Fisher Scientific) and 1% l-Glutamine (2 mM, Sigma-Aldrich, St. Louis, MO, USA) and 1% Pen-Strep (100 U/mL, Sigma-Aldrich, St. Louis, MO, USA). The cell line was maintained at 37 °C with 5% CO_2_.

NAC (N-Acetyl-l-cysteine, A0150000, Sigma-Aldrich, St. Louis, MO, USA) treatments were performed utilizing two protocols. The former consisted of the treatment with NAC 5 mM for 24 h of infected and control (uninfected) cells before thiol evaluations (co-NAC label), which simulates a therapeutic intervention with this Cys analogue that starts at the beginning of the infection (i.e. therapy mode). During this treatment the cells were grown in a culture medium deprived of sulfur-containing amino acids (DMEM, high glucose, no methionine, no cystine, 21013024, Gibco, Thermo Fisher Scientific); these conditions are identified with the label NCM, i.e. non-complete medium. This latter is a strategy adopted to obtain more reliable measurements of Cys and other cellular thiols released in the extracellular environment [24]. The other protocols consisted of a pre-treatment for 24 h of the cells with NAC in complete medium (CM) and then the study of thiols after 24 h from the infection in which the cells were maintained in NCM without NAC (pre-NAC label). With this treatment protocol the effects of NAC were investigated in chemoprevention mode.

Remdesivir (Rem; MedChem Express, NJ, USA, HY-104077), Nelfinavir (Nel; AMBH2D6EF522, Sigma-Aldrich, St. Louis, MO, USA), Saquinavir mesylate (SM, Sigma-Aldrich, St. Louis, MO, USA), Indinavir Sulfate (IS, Sigma-Aldrich, St. Louis, MO, USA) and Ebs (E3520, Sigma-Aldrich, St. Louis, MO, USA) were dissolved in dimethyl sulfoxide (DMSO, Sigma-Aldrich, St. Louis, MO, USA) and aliquots of concentrated stock solutions were maintained at −80 °C until further use. During cellular treatments the compounds were diluted at the indicated concentrations in cell culture medium to obtain final DMSO concretions <0.001%.

Cytotoxicity and cell viability during treatments were assessed using MTT assay according to Ref. [25].

### 2.3 SARS-CoV2 yield reduction assay

One day prior to the experiment, 20,000 cells/well were seeded in 96-well flat-bottom plates. Cells were infected with SARS-CoV2 at a multiplicity of infection (MOI) of 0.0035 (50 TCID50/well) in complete medium for 1 h and then antiviral compounds were added and incubated at 37 °C in a humidified incubator with 5% CO_2_. Uninfected (mock) controls were included in each plate. At 48–72 h post-infection, cells were fixed with 10% (v/v) neutral formalin (Leica) and stained with crystal violet (Sigma-Aldrich, St. Louis, MO, USA). Cell viability and cytotoxicity were assessed in parallel, in identically treated, uninfected plates. Three independent experiments were performed, each including a technical duplicate.

### 2.4 Plaque-reduction assays

Vero-E6 cells (450,000 cells/well) were seeded in a 6-well plate and incubated at 37 °C with 5% CO_2_ for 24 h. After incubation, the medium was removed and cells were infected with 500 μl of ten-fold serial dilution of SARS-CoV2 stock for 1 h, rocking plates every 15 min. In the meanwhile, the overlay medium (complete medium with agar 0.1%) was prepared and maintained in a 50 °C water bath. Subsequently, different doses of Nel or Rem in 1.5 ml of complete medium were added and incubated for 30 min. The overlay medium (2 ml) was poured into each well and the plates incubated for 3 days.

Finally, the overlay was discarded, cells were fixed for 30 min with 4% paraformaldehyde (PFA) and stained with crystal violet working solution. Viral titer was determined as plaque-forming units per ml, considering wells with plaques ranging from 2 to 100. All experiments performed in three biological replicates.

### 2.5 RT-qPCR analysis of SARS-CoV2 RNA

RNA was extracted from SARS-CoV2 infected and uninfected cells using the QIAsynphony DSP Virus/Pathogen kit (Qiagen GmbH, Hilden, Germany), according to the manufacturer's instructions and used in RT-qPCR assays along with a standard curve generated using serial dilutions (10^4^, 10^3^, 10^2^ and 10 viral copies/reaction) of the RNA positive control provided by the TaqPath COVID-19 RT-PCR kit (applied biosystems, TermoFisher Scientific Life Technologies Corp. Pleasanton, CA, USA). Primer/probe sets used in RT-qPCR assays targeted the S, N and ORF1ab genes. The RT-PCR was performed on a 7500 Fast Dx RT-PCR instrument (ThermoFisher), under the following conditions: retro-transcription was carried out by sequential incubation at 25 °C for 2 min, 53 °C 10 min, and 95 °C 2 min, followed by 40 cycles of denaturation at 95 °C for 3 s, extension and collection of the fluorescence signal at 60 °C for 30 s. The corresponding viral genome copy number to each Ct value was calculated based on the standard curve.

### 2.6 Immunoblotting assay

VERO-E6 cells were prepared for immunoblot analysis as described in [26]. Proteins were quantified in the cellular extracts by the bicinchoninic acid (BCA) assay using bovine serum albumin as an external standard [27]. Proteins (20 μg) were loaded onto 4–12% sodium dodecyl sulfate–polyacrylamide gel electrophoresis (SDS–PAGE) minigels (Novex WedgeWell Tris-Glycine gel, Invitrogen), and immobilized on nitrocellulose membrane for immunoblot analysis. The membranes were then incubated with 5% skim milk in Tris-buffered saline (TBS; 20 mM Tris base, 150 mM NaCl, pH 7.4) and 0.1% Tween-20 for 2 h at room temperature. The blots were incubated with primary antibodies at 4 °C overnight, with constant shaking and then washed twice with TBS. The primary antibodies were: anti-xCT (ab175186; 1:1000) from Abcam, anti β-actin (#3700; 1:1000), anti-GAPDH (#5174; 1:1000), anti-PERK(C33E10) (#3192; 1:1000), anti-Nrf2 (#12721; 1:1000) from Cell Signaling Technology, anti-MRP1 (QCRL-1) (sc-18835; 1:500) from Santa Cruz Biotechnology, Inc., and anti-GCLC (E-AB-52359, 1:2000) from Elabscience. The secondary antibodies were anti-rabbit (#7074) or anti-mouse (#7076) IgG 1:2000) horseradish peroxidase-linked (Cell Signaling Technology). Protein bands were detected using an ECL Clarity Max (BioRad). Quantification of bands was performed with a Gel-Pro Analyzer; protein expression level was normalized to housekeeping protein expression.

### 2.7 Protein glutathionylation (PSSG)

To produce reliable qualitative and semiquantitative results, PSSG determinations were carried out with three different procedures, including HPLC, immunoblot, and Immunofluorescence analysis. After alkylation of Cys residues with NEM [28], cellular proteins (20 μg) were fractionated under non-reducing conditions in 4–12% gradient SDS–PAGE and then transferred to a nitrocellulose membrane (Millipore) for immunoblot analysis utilizing an anti-GSH primary antibody (1:1000, Abcam) and an anti-mouse IgG HRP-linked secondary antibody (1:5000, Cell Signaling Technology). Immunofluorescence analysis of PSSG was carried out using the Cayman's Glutathionylated protein detection kit (Cayman Chemical, Item No.10010721) on Operetta CLS (PerkinElmer). HPLC analysis of PSSG was performed as described in Ref. [28].

### 2.8 Thiols and disulfide analysis

Total cellular thiols (including both low-MW and protein thiols) were preliminarily assessed utilizing the Ellman reagent (5,5′-dithiobis-2- nitrobenzoic acid; Sigma-Aldrich; D8130) suspended in PBS, pH 7.5, to a final concentration of 200 μM 100 μg of test sample proteins were incubated with the reagent for 5 min at 37 °C, and then the absorbance was measured at 412 nm. Cellular thiol concentrations were calculated against a calibration curve of GSH (Sigma-Aldrich).

Individual species of low molecular mass thiols and disulfides were assessed in Vero E6 cells by HPLC analysis with fluorescence detection after derivatization with monobromobimane (mBrB, Calbiochem) [24,28,29]. Extracellular thiols were measured as the sum of low molecular mass thiols and disulfides, and protein-bound thiols after sample reduction with dithiothreitol (DTT, Sigma-Aldrich) and labelling of the released thiols with mBrB. For intracellular thiol and disulfide analysis cells were seeded at a density of 450x10^3^ cells/well in 6 well plates. After treatment and/or infection, the culture medium was removed and the cells were washed twice with phosphate buffered saline solution pH 7.4 (PBS) containing (for disulfide detection) or not (for thiol detection) 5 mM N-ethylmaleimide (Sigma-Aldrich) to block reduced species. Cell lysis was carried out in 0.5 ml of 4% (w/v) trichloroacetic acid (TCA) by gentle scraping. Thiols detected by immediate labelling with mBBr whereas disulfides were reduced first with DTT and then labelled with mBrB.

### 2.9 Intracellular protein concentration

Protein acidified pellets were resuspended in 0.2 ml of 0.1 N NaOH. Determination of protein concentration was performed by the Bradford assay [30]. Bovine serum albumin (BSA) was used as standard.

### 2.10 Immunofluorescence assay and Operetta CLS quantitative fluorescence analysis

After infection and/or treatment, Vero E6 cells in 96 well plates flat bottom black polystyrene wells (with micro-clear bottom, Greiner CELLSTAR®) were fixed with 10% neutral formalin (Leica), and then washed in three times with PBS (Euroclone) and permeabilized with Triton X-100 0,5% in PBS for 5 min at room temperature (RT). Subsequently, blocking was carried out with 3% FBS for 15 min at RT under slow stirring. At the end of the blocking, a washing was carried out with distilled water and then the incubation with the primary antibody was carried out in 3% FBS for 1 h at RT and under slow stirring. Then, 3 washes were carried out in distilled water and the labelled secondary antibodies diluted 1: 1000 and the DAPI (Life Technologies) or Hoechst 33,342 (Enzo Life Sciences) to stain the nuclei (1: 3000) were placed in 3% FBS for 1 h in the dark. Afterward, 4 washes were carried out in distilled water and were added phalloidin-Alexa Fluor 595 or Alexa Fluor 488 (Life Technologies) to stain actin filaments (1: 1000 in PBS) for 40 min in the dark under stirring. Finally, 6 washes were carried out in sterile distilled water (Euroclone) and the black plates (Greiner CELLSTAR®) were ready for quantitative fluorescence analysis (from 63 to 72 fields/well) and image acquisition with 40X and/or 63X objectives with water system by Operetta CLS system (PerkinElmer). The primary antibodies used were: Nrf2 Polyclonal Antibody, FITC Conjugated (bs-1074R-FITC, Bioss Antibodies, 1:50); NQO1 (A180, Mouse mAb #3187, CST, 1:50); GST-pi (610,718, mouse mAb, BD Biosciences, 1:500); PE anti-human IL-6 Antibody (BioLegend, 1:1000); PE anti-human IL-10 Antibody (BioLegend, 1:1000). The secondary antibodies used were: Texas Red anti-rabbit IgG and Cy5 anti-mouse IgG (Invitrogen).

### 2.11 Statistical analysis

The calculated means of all measurements were compared for statistical significance by One-Way ANOVA followed by Tukey's test for multiple comparison or Student's *t*-test. Statistical analysis was performed using GraphPad Prism (v.6.0).

## 3 Results

### 3.1 Levels and NAC-stimulated metabolism of GSH and other cellular thiols in SARS-CoV-2 infected cells

Cellular thiols were investigated in Vero E6 cells 24 hpi; according to previous findings in literature [4,5], this time point in the SARS-CoV2 infection process precedes the phase in which CPE can be detected by morphology analysis and cell viability tests (measured 48 hpi), and plaque assay (72 hpi) (Suppl. Figure 1). Other molecular changes observed 24 hpi included chromatin condensation and modifications of cellular IL-6 and IL-10 levels (Suppl. Figure 1).

**Fig. 1:**
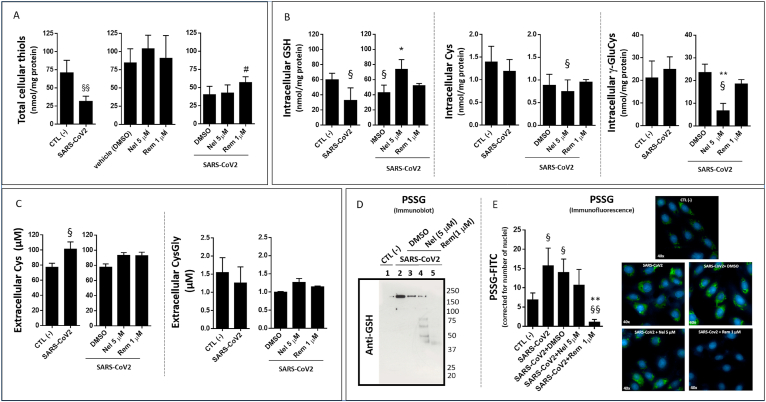
**Levels of thiols and protein glutathionylation in SARS-CoV2 infected VERO E6 cells treated with Remdesivir (Rem) and Nelfinavir (Nel) antiviral compounds.** The cells were grown in complete medium and infected with SARS-CoV2 (MOI:0.0035) as described in the section Methods. Treatments with Rem and Nel utilized at the final concentrations of 1 μM and 5 μM (in DMSO, 0.001% vol/vol) lasted 24 hpi. Total intracellular thiols **(A)** were measured by Ellman's test using 100 μg of protein extract of VERO E6 cells. Intracellular **(B)** and extracellular **(C)** levels of GSH and other thiols measured by HPLC 24 hpi. in cells treated with Rem or Nel. S-glutathionylation (PSSG) of cellular proteins assessed 24 hpi by immunoblot **(D)** or by semi-quantitative immunofluorescence (PSSG-FITC, green; DAPI for nuclei, blue) **(E)**. Control test with untreated cells (CTL -) *vs* infected cells or treatments: §p < 0.05, §§p < 0.01. Infected cells + DMSO *vs* antivirals: *p < 0.05, **p < 0.001.

As a main finding in this study, SARS-CoV2 infection significantly decreased the levels of total cellular thiols (Fig. 1A) and intracellular GSH (Fig. 1B), whereas the extracellular levels of Cys increased (Fig. 1C) in comparison with uninfected control cells.

**Fig. 2:**
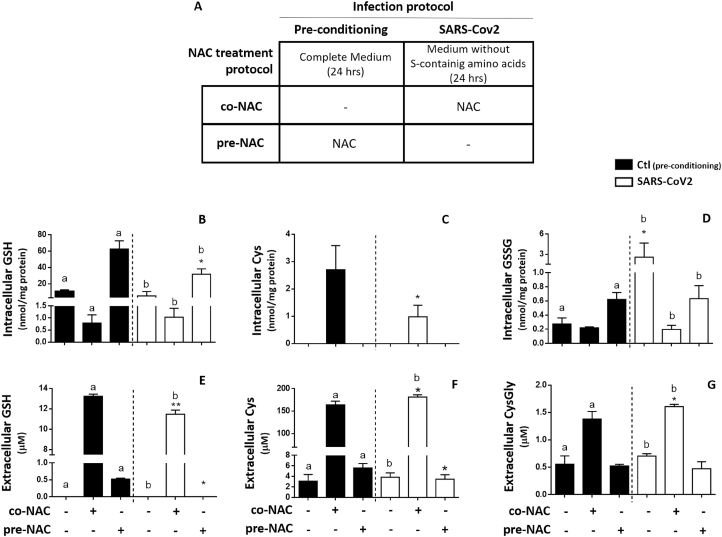
**Effect of NAC on cellular thiols of SARS-CoV2 infected VERO-E6 cells.** VERO E6 cells were treated with 5 mM NAC utilizing two different protocols: in the pre-NAC protocol the cells were treated in complete medium (CM) with NAC before infection and then NAC-containing medium was replaced with a medium deprived of sulfur-containing amino acids (non-complete medium or NCM) for infection; in the co-NAC protocol the cells were directly exposed to NAC in NCM during the infection **(A)**. Thiols were assessed 24 hpi. intracellular GSH **(B)**; intracellular Cys **(C)**; Intracellular GSSG **(D)**; extracellular GSH **(E)**; extracellular Cys **(F)** and extracellular CysGly **(G)**. NCM *vs* co-NAC or pre-NAC: **a** (control test, black bars) and **b** (SARS-CoV2 infected cells, white bars): p < 0.05; control cells (black bars) *vs* SARS-CoV2 infected cells (white bars) in the same NAC treatment conditions: *p < 0.05; **p < 0.001.

These changes of SARS-CoV2 infected cells, were even more evident during the stimulation of GSH metabolism with NAC (Fig. 2). Treatments with this Cys analogue were performed utilizing the protocols described in Fig. 2A and included administrations of NAC carried out either before (chemoprevention mode) or during the exposure to the virus (treatment mode). The two modalities identified as pre-NAC and co-NAC protocol, respectively, had in common the removal of sulfur-containing amino acids from the culture medium (which is identified as non-complete medium or NCM) during the second step of the treatment in which the cells were inoculated with the virus. Although removing Cys and Met from the cell culture medium modifies the physiological control of cellular thiols (Suppl. Figure 2), this is a forced step to avoid interferences of these thiols with NAC that was utilized for the cell treatments. At the same time, this manipulation of medium composition offers more reliable and sensitive measurements of the efflux of cellular thiols [24].

As far as NAC treatment is concerned, SARS-CoV2 infected cells showed a lowered response to the pre-NAC protocol compared to control uninfected cells, with reduced intracellular levels and efflux of GSH (Fig. 2B and E, respectively), and decreased extracellular levels of Cys (Fig. 2F).

On the other hand, SARS-CoV2 infection reduced the effect that the co-NAC protocol produced on the Cys levels of control cells (Fig. 2C) also reducing the stimulation effect that this protocol produces on the efflux of cellular GSH (Fig. 2E). At the same time, the infected cells showed increased extracellular levels of Cys and CysGly compared to control cells in response to co-NAC treatment (Fig. 2F and G, respectively).

To summarize these findings, SARS-CoV2 infection alters the balance of thiols found at the two sides of the cellular membrane leading to a marked decrease of cellular thiols, and especially of GSH (by more than 50% of the control levels, Suppl. Table 2). The limited response to NAC indicates that such defect of cellular thiols can be explained by a reduced capability of the infected cell to sustain the de novo biosynthesis of GSH.

**Fig. 3:**
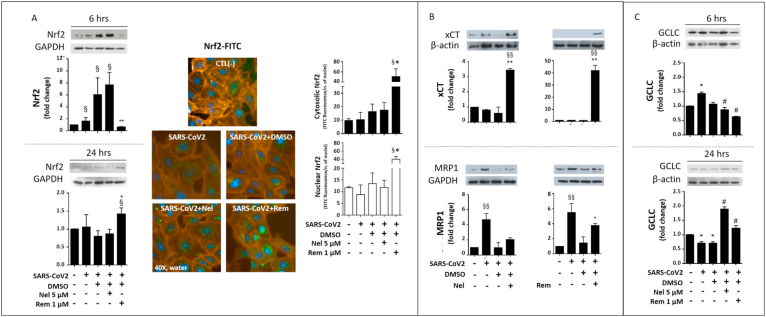
**Nrf2, Membrane transporters, and GCLC in SARS-CoV2 infected VERO-E6 cells treated with Nelfinavir (Nel) or Remdesivir (Rem).** Immunoblot of Nrf2 protein expression (**A**, left panels) was assessed 6 hpi and 24 hpi, and by semi-quantitative fluorescence analysis 48 hpi (**A**, right panels). Fluorophores were FITC (green) for Nrf2 protein labelling, DAPI (blue) for nuclei and Phalloidin-Alexa Fluor595 (orange) for the cytosolic space. Immunoblot of xCT and MRP1 membrane transport proteins (**B**), and GCLC protein (**C**) were carried out as described in the section Methods 24 hpi. Infection conditions and treatments with antivirals were the same of Fig. 1. Control test with untreated cells (CTL -) *vs* infected cells or treatments: §p < 0.05, §§p < 0.01. Infected cells + DMSO *vs* antivirals *p < 0.05, **p < 0.001. (For interpretation of the references to colour in this figure legend, the reader is referred to the Web version of this article.)

**Fig. 4:**
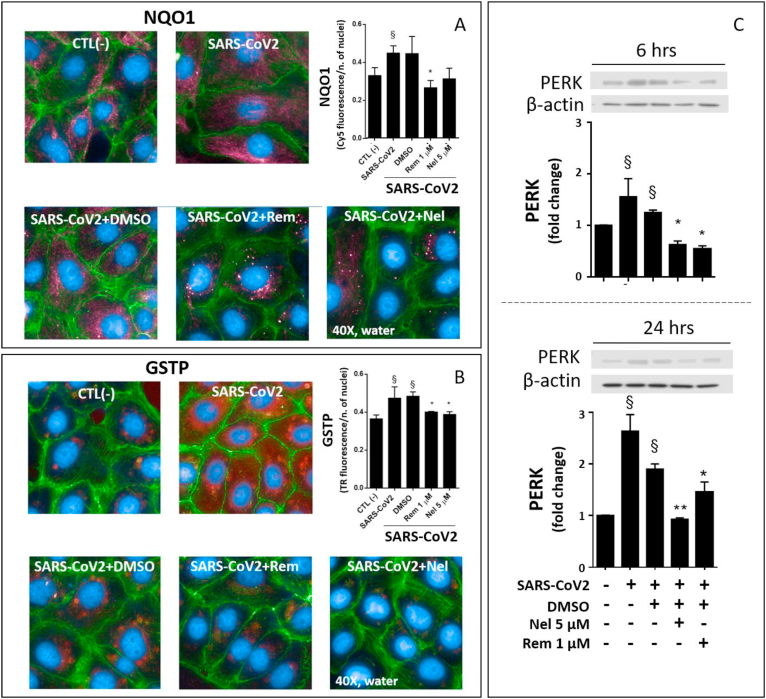
**Expression of the stress proteins NQO1, GSTP and PERK in SARS-CoV2 infected VERO-E6 cells treated with Nelfinavir (Nel) or Remdesivir (Rem).** Qualitative and semi-quantitative fluorescence analysis of NQO1-Cy5 (red) **(A)** and GSTP-Texas Red (red) **(B)** were measured 24 hpi. Nuclei were labelled with DAPI (blue) and cytosol with Phalloidin -Alexa Fluor488 (green). Immunoblot of PERK protein expression was assessed by immunoblot 6 hpi and 24 hpi **(C)**. Infection conditions and treatments with antivirals were the same of Fig. 1. Control test with untreated cells (CTL -) *vs* infected cells or treatments: §p < 0.05, §§p < 0.01. Infected cells + DMSO *vs* antivirals *p < 0.05, **p < 0.001. (For interpretation of the references to colour in this figure legend, the reader is referred to the Web version of this article.)

To further explore the GSH depletion mechanism induced during SARS-CoV2 infection, the expression of two membrane transporters with possible role in the defective availability of cellular Cys was investigated. In comparison with control cells, the infected cells showed a trend toward a lowered expression of xCT (Fig. 3B), a protein active in Cyss uptake [31]. Whereas the phase 3 drug metabolism gene MPR1 that is involved in thiol efflux during the response to cellular stressors [10,13,24], was significantly upregulated by the infection (Fig. 3B). The infection also up-regulated the detoxification and drug metabolism proteins NQO1 and GSTP (Fig. 4A and B, respectively). The expression of all these proteins depend on highly inducible genes with key role in the cellular stress response to cellular electrophiles. These genes are regulated by different transcriptions factors, including Nrf2 [13,32]. However, Nrf2 protein expression and its nuclear translocation for transcriptional activation were not affected during the different phases of the infection investigated in this study (Fig. 3A and Suppl. Figure 3).

### 3.2 Glutathione redox, protein glutathionylation and stress response pathways

Besides to GSH depletion, SARS-CoV2 infection markedly stimulated the oxidation of cellular glutathione to its disulphide form (GSSG) (Fig. 2D and Suppl. Figure 2C); an increased oxidation of cellular Cys and γ-GluCys to the corresponding disulphides was also observed (Suppl. Table 2).

Important enough was the finding that NAC, independent of treatment modality (prevention mode or therapy mode, Fig. 2A), was effective in reducing the levels of glutathione oxidation observed in SARS-CoV2 infected cells (Fig. 2D).

Also, the levels of protein glutathionylation (PSSG) increased in SARS-CoV2 infected cells compared to control cells (Fig. 1D), and a characteristic cytosolic localization was observed for this post-translational modification of the cellular proteins (Fig. 1E).

These results demonstrate that the SARS-CoV2 infection promotes a pro-oxidant environment in the host cell, ultimately affecting the redox of both free and protein-bound Cys residues. According with a role of PSSG formation in the modulation of stress pathways, including ER stress [22], the protein levels of PERK were upregulated starting from 6 hpi (Fig. 4C), and an increased expression was also observed for the detoxification and stress response genes described earlier in Section 3.1, including MRP1, GSTP and NQO1 (Figs. 3B and Fig. 4A–B).

### 3.3 The effect of antiviral drugs on the GSH impairing effect of SARS-CoV2 infection

The CPE, viral RNA load and inflammatory cytokines in SARS-CoV2 infected cells were all significantly reduced by the treatment with the FDA-approved HIV-1 protease inhibitor Nel and to a lower extent by the nucleotide analogue Rem, which has been investigated in the antiviral therapy of COVID-19 [33] (Suppl. Figure1). Almost no antiviral activity was observed for the other HIV-1 protease inhibitors Indinavir sulfate and Saquinavir mesylate (Suppl. Fig. 1, A-B). Based on these findings, Nel and Rem were selected to explore the effect of their antiviral function on cellular thiols and CPE process of the infected cells.

Not only Nel was the most potent antiviral agent among those investigated in this study, but it also showed potent GSH enhancing activity (Fig. 1B). Such GSH enhancing effect of Nel was associated with reduced cellular levels of Cys and particularly of γ-GluCys (Fig. 1B), suggesting higher utilization of these thiols in the de novo biosynthesis of GSH. On the other hand, Rem treatment of SARS-CoV2 infected cells, showed lower capability to stimulate cellular GSH, and did not affect the levels of the GSH biosynthesis precursors Cys and γ-GluCys (Fig. 1B). This latter finding may explain the highest levels of total thiols measured with the Ellman's method in infected cells treated with this antiviral agent (Fig. 1 A).

The different efficacy of Nel compared to Rem in stimulating the cellular metabolism of GSH was also confirmed when the availability of Cys was increased in the infected cells by NAC treatment (pre-NAC protocol) (Suppl. Figure 4).

At the molecular level, the higher GSH enhancing properties of Nel compared to Rem, found mechanistic support in an earliest induction of Nrf2 protein expression (6 hpi data shown in Fig. 3A) that was associated with changes in the expression of membrane transporters (xCT upregulation and MDR1 inhibition; Fig. 3B) and glutamate-cysteine ligase catalytic subunit (GCLC) (Fig. 3C) observed 24 hpi. These molecular findings are all coherent with thiol analysis data (presented earlier and in Fig. 1 and Suppl. Figure 4) indicating higher availability and utilization of cellular thiols for GSH synthesis in Nel-compared to Rem-treated cells.

However, immunoblot data suggest that Rem markedly stimulated the expression of the redox-sensitive transcription factor Nrf2 (Fig. 3A, **left panels**), but this effect occurred late in the cell infection process, i.e. from 24 hpi onward, and a significant nuclear translocation of Nrf2 was observed analysis 48 hpi only (Fig. 3A, **right panels** vs. Suppl. Figure 3). This nuclear translocation process is required for this transcription factor to bind ARE/ERE sequences in the promoter region of antioxidant and detoxification proteins, which was confirmed in infected cells treated with Rem by the up-regulation of the Nrf2-dependent protein xCT (Fig. 3B), but not of NQO1 and GSTP proteins that decreased after treatment (Fig. 4A and B, respectively).

Furthermore, Rem treatment was very efficient in reducing PSSG formation in the infected cells (Fig. 1D–E), whereas Nel treatment demonstrated lower efficacy (Fig. 1D and F). .

Also, the alkylating (thiol-reacting) compound Ebs [13,24] with proposed activity as viral protease inhibitor [34], significantly enhanced the cellular levels of GSH in SARS-CoV2 infected cells (Suppl. Fig. 5A, C), but this effect was associated with increased levels of the oxidized form of glutathione, i.e. GSSG (Suppl. Figure 5B), which is expected from the pro-oxidant properties of this compound [24].

## 4 Discussion

Infections with respiratory viruses induce cytopathic effects sustained by the viral replication process itself and by the occurrence of characteristic alterations of the cellular redox [35]. A depletion of the cellular antioxidant glutathione has convincingly been demonstrated to play a role in this context [22].

SARS-CoV2 infection of Vero E6 cells, recapitulates these alterations that have been characterized for the first time in this study as far as their biochemical and molecular specificities are concerned (summarized in the scheme of Suppl. Figure 6). Mechanistically, SARS-CoV2 infection decreases the levels of cellular thiols, and especially of GSH (Fig. 1 and Suppl. Table 1); apparently, the infection reduces the cellular availability of Cys and consequently the potential of the host cell to sustain the de novo biosynthesis of GSH. This observation was confirmed by the stimulation of GSH metabolism with the Cys analogue NAC administered to Vero E6 cells in therapy mode, i.e. during the exposure to the virus (co-NAC protocol, Fig. 2). This modality of treatment was also useful to demonstrate that the infection reduces the efflux of the newly synthesized GSH in the extracellular milieu (Fig. 2E). This latter evidence and the presence of increased levels of extracellular Cys and CysGly in infected cells treated with the co-NAC protocol (Fig. 2F and G), support the view that the SARS-Cov2 infection also interferes with the homeostatic control of extracellular thiols, which may increase the infection potential of the virus [21].

In keeping with mechanistic aspects of these alterations of cellular and extracellular thiols in SARS-CoV2 infected cells, these were associated with a modified expression of proteins involved in the transmembrane fluxes of Cys and other cellular thiols. These include the cystine transporter xCT [31], the expression of which decreases by the infection. On the other hand, the membrane transporter MRP1 was markedly upregulated. MDR1 is one of the transporters involved in the efflux of cellular thiols, a process that redistributes GSH and other thiols at the two sides of the membrane during the response to cellular stressors with possible role in the commitment and execution phase of the apoptotic program (described in Refs. [10,24] and references therein).

The depletion of cellular GSH in Sendai as well as in other viral infections, including HIV and influenza, has repeatedly been associated with increased mixed disulfide formation on cellular proteins (reviewed in Ref. [22]). Increased levels of this type of post-translational modification were also observed in SARS-CoV2 infected cells. A concomitant increase of the non-protein disulphides GSSG, Cyss and γ-GluCyss confirms the view that PSSG formation is the result of an unbalanced cellular redox and insufficient availability of cellular GSH to prevent unspecific protein Cys thiolation. At the same time, GSTP protein levels increased upon SARS-CoV2 infection. Available data support the view that this is a main enzymatic player of protein glutathionylation reactions induced by different types of cellular stressors and adverse conditions, including reactive oxygen species, alkylating agents and Cys shortage [24,36]. At the same time, glutaredoxins, thioredoxin reductase and other oxidoreductases are involved in deglutathionylation pathways [12]. To our knowledge, this is the first evidence of an increased expression of GSTP induced by a viral infection, whereas defects in de-thiolation processes have already been evoked to explain the altered protein glutathionylation and redox signaling of cells infected with different types of viruses including influenza, HIV and HSV (reviewed in Ref. [22]). In HIV-infected T lymphocytes, deglutathionylation appears to be slower than in uninfected cells, and NAC has been reported to correct such defect [37].

Under these circumstances, PSSG formation is reported to sustain both ER stress and the activity of other important pathways with specific role in the adaptive response to cellular stressors, such as Nrf2, autophagy and programmed cell death (extensively reviewed in Refs. [9,12,22,32,[38], [39], [40]]). Therefore, our findings are consistent with the hypothesis that GSH depletion may have a direct role in the abnormal protein redox and consequently in the accumulation of protein damage as a possible cause of ER stress in SARS-CoV2 infected cells. However, we cannot exclude the presence of other mechanisms of UPR and ER stress signaling. In fact, similarly to other viruses, pathogenic aspects of SARS-CoV2 infection observed at cellular level include an abnormal synthesis and accumulation of viral proteins in the endoplasmic compartment during the earliest phases of virus replication [22]. SARS-CoV proteins, such as the E protein, may have a direct role in ER stress and in the apoptotic signaling of the infected cell, as well as in the release of the matured virus by the formation of the ER–Golgi intermediate compartment [41,42]. In our study, the ER stress response to SARS-CoV2 infection was demonstrated by PERK expression analysis. The upregulation of this protein is a very early event in the infection process already present 6 hpi, which is in agreement with previous findings obtained in HSV [16] and other types of viral infections [22]. These pieces of evidence demonstrate the difficulty of developing efficient and timely strategies of cytoprotection for this infection.

In this context, GSH enhancing strategies and the prevention of protein glutathionylation may hold great potential as antiviral strategies [17,21]. A series of UPR inhibitors, including NAC [43], are already under investigation as endothelial cell protection agents [44] and antivirals in the context of COVID-19 [45,46]. NAC has also been utilized to replenish blood cell glutathione in HIV-infected patients, leading to improved immune function, drug metabolism and protection against oxidative stress [20].

In this study we demonstrated that efficient antiviral drugs, such as the viral protease inhibitor Nel, provide another, and very efficient, way to restore cellular GSH in SARS-CoV2 infected cells (Fig. 1 and Suppl. Figure 4). The in vitro efficacy of this drug to inhibit viral replication in Vero-E6 cells was already described in literature [47], but its GSH enhancing properties were unknown. Nel (Viracept) has been utilized for the treatment of HIV in association with other drugs, but its production has been suspended in 2012 due to the advent of other and more efficient therapies with better efficacy and toxicity profile (a significant drug toxicity was also confirmed in our in vitro study, Supplementary Table 3). Mechanistically, the effect of Nel treatment on cellular GSH appears to depend on a transient stimulation of Nrf2 expression that occurs early in the infection process (6 hpi) to induce the expression of genes with main role in GSH biosynthesis, such as GCLC and the membrane transporter xCT (Fig. 3); such a timely response was associated with the blockade of viral replication and CPE (Suppl. Figure 1), and with the inhibition of ER stress signaling (Fig. 4C). Moreover, a synergistic effect with NAC in stimulating the GSH metabolism of infected cells was observed for this antiviral drug (Suppl. Figure 4), holding potential for combinatorial protocols with redox modulators and UPR inhibitors that, beside NAC, may include other Cys and GSH analogues [17,21,48].

Conversely, the nucleotide analogue Rem originally developed for treating Ebola virus, showed much lower antiviral activity in Vero-E6 cells (confirmed in Ref. [47]) and capability to stimulate the biosynthesis of cellular GSH compared with Nel. Notwithstanding, Rem was a potent Nrf2 activator and inhibitor of the SARS-CoV2 induced formation of cellular PSSG, but these effects of Rem occurred later in the infection process (from 24 hpi onward) compared with Nel (6 hpi), which may explain the limited cytoprotection activity of this antiviral in our in vitro model of SAR-CoV2 infection, as well as the disappointing results obtained in COVID-19 therapy [49].

A GSH enhancing activity in SARS-CoV2 infected VERO E6 cells was also demonstrated for the antiviral agent Ebselen [34]. Such an effect is expected from the alkylating and *para*-hormetic properties of this seleno-organic compound [13,24]. The increase cellular levels of GSSG observed during the treatment with this compound, confirm these properties. On the other hand, this type of response may represent a concern because SARS-CoV2 infection itself interferes with the redox hemostasis of cellular thiols, and this has been speculated to represent a common mechanism of viral infections to promote a pro-oxidant environment in the host cell which is conductive to viral replication [22,35].

In conclusion, SARS-CoV2 infection impairs the metabolism of cellular GSH and its role in the redox homeostasis of cellular proteins. These alterations may contribute to the UPR and ER stress of the host cell, which are key steps in the CPE of SARS-CoV2 and many other types of viruses. Other consequences appear to include changes in the composition of extracellular thiols that deserve further investigation for possible roles in SARS-CoV2 infection [21]. Antiviral drugs can be utilized to prevent the changes of cellular thiols observed during the infection, at least to some extent. Mechanistic aspects standing behind this effect support a causal link between an improved biosynthesis of GSH and a timely control of the CPE in the host cell by blocking virus replication, that is in agreement with previous findings obtained with other antiviral agents and viral infection models [17]. Compound-specific differences in the modulation of cellular GSH and protein glutathionylation in the infected cell worth investigating to develop more efficient strategies of cytoprotection in SARS-CoV2 infection.

A limit of this study is the utilization of VERO E6 cells instead of a cell model that may more closely represent the lung cell targets of SARS-CoV2. However, the VERO E6 cell line remains the most reliable and biologically relevant model to assess the SARS-CoV2/host cell interaction [50], and it has recently been utilized to characterize in great detail the CPE of SARS-CoV2 as far as infection kinetics and morphological aspects are concerned [4,5]. The Vero cell model has also been utilized to characterize the pharmacological properties of Rem and Nel; the latter is a protease inhibitor previously utilized in HIV patients that similarly to other protease inhibitors active on SARS-CoV and SARS-CoV2 infection of human and animal epithelial cells [51], has been confirmed to be an efficient antiviral agent in SARS-CoV2 infected Vero E6 cells [52]. These pharmacological and cellular studies have been utilized to design our in vitro investigation and it is important to remark that the overall CPE process of this cell line shows a substantial correspondence of with that of human cell lines or primary cells isolated from the respiratory tract [53,54], thus evoking the pathological features of the lung tissue of COVID-19 patients that involve pneumocytes and type II cells [55,56]. Furthermore, SARS-CoV2 has the potential to infect several human ACE2 expressing cells [51], which include kidney epithelial cells.

## Funding

This work was supported by “10.13039/501100004486Fondazione Cassa di Risparmio di Perugia” [grant number: 19,837 (2020.0522)]

## Declaration of competing interest

The authors declare no competing financial or non-financial interests.
