# Supplementary material for: SARS-CoV2 infection impairs the metabolism and redox function of cellular glutathione
Source: Redox Biol. 2021 Jun 10;45:102041. doi: 10.1016/j.redox.2021.102041 (PMC8190457; doi:10.1016/j.redox.2021.102041)
Supplement: Multimedia component 1 [file mmc1.docx]

**Supplementary material:**

- **suppl. Figure 1-6**
- **suppl. Table 1-3**

Title:

SARS-CoV2 infection impairs the metabolism and redox function of cellular glutathione.

Authors:

Desirée Bartolini 1,2; Anna Maria Stabile 2; Sabrina Bastianelli 3; Daniela Giustarini 4; Sara Pierucci 3; Chiara Busti 3; Carmine Vacca 5; Anna Gidari 3; Daniela Francisci 3; Roberto Castronari 6, Antonella Mencacci 6; Manlio Di Cristina 5; Riccardo Focaia 5, Samuele Sabbatini 7, Mario Rende 2; Antimo Gioiello 1; Gabriele Cruciani 5; Ranieri Rossi 4; Francesco Galli 1,*.

Affiliations:

1 Department of Pharmaceutical Sciences, University of Perugia, Nutrition and clinical biochemistry lab, Via del Giochetto, Monteluce, Perugia, Italy

2 Department of Medicine and Surgery, Section of Human, Clinical and Forensic Anatomy, School of Medicine, University of Perugia, P.le Lucio Severi, 1, Sant’Andrea delle Fratte, 06132 Perugia, Italy

3 Department of Medicine and Surgery, Clinic of Infectious Diseases, University of Perugia, Perugia, Italy

4 Department of Biotechnology, Chemistry and Pharmacy, University of Siena, Via A. Moro 2, I-53100 Siena, Italy.

5 Department of Chemistry, Biology and Biotechnology, University of Perugia, Via Elce di Sotto 8, 06123 Perugia, Italy

6 Department of Medicine and Surgery, Microbiology Unit, University of Perugia, 06123 Perugia, Italy

7 Department of Medicine and Surgery, Medical Microbiology Section, University of Perugia, 06129 Perugia, Italy

*corresponding author:

Galli Francesco, PhD

Applied Biochemistry and Nutrition Lab

Dept. of Pharmaceutical Sciences, University of Perugia

+39 0755857445/90; +39 3356858596

francesco.galli@unipg.it

**Supplementary Figure 1. Cytopathic effect and activity of antivirals in SARS-CoV2 infected VERO E6 cells.**

VERO-E6 cells were infected with SARSCOV2 (MOI = 0.0035) and CPE was assessed 48 hpi by crystal violet staining **(A)** and direct microscopic analysis **(B)**. Treatments with antiviral agents started 1 hpi and included Nelfinavir (Nel), Remdesivir (Rem), Saquinavir Mesylate (SM) and Indinavir Sulfate (IS). The antiviral effect of Nel **(C)** and Rem **(D)** treatments (at the indicated concentrations and in DMSO) was investigated 72 hpi with plaque assay in 6-well plates utilizing a 10^-5^ dilution (**C** and **D**) or serial dilutions from 10^-2^ to10^-6^ of SARS-CoV2 (3.16 ×10^7^ TCID50/mL) **(E)**. Chromatin condensation was assessed as an indicator of cellular damage 24hpi by DAPI staining of nuclei **(F)**. Intracellular levels of IL-6-PE **G)** and IL-10-PE **H)** were measured 24hpi by semiquantitative fluorescence analysis of cells. Viral RNA copy numbers in the cell supernatants were assessed 48hpi for Nel and Rem treatments by real-time RT-PCR **(I)**. Control test with untreated cells (CTL -) *vs* infected cells or treatments: § p<0.05, §§ p< 0.01. Infected cells + DMSO *vs* antivirals *p<0.05, **p<0.001; ***p<0.0001.


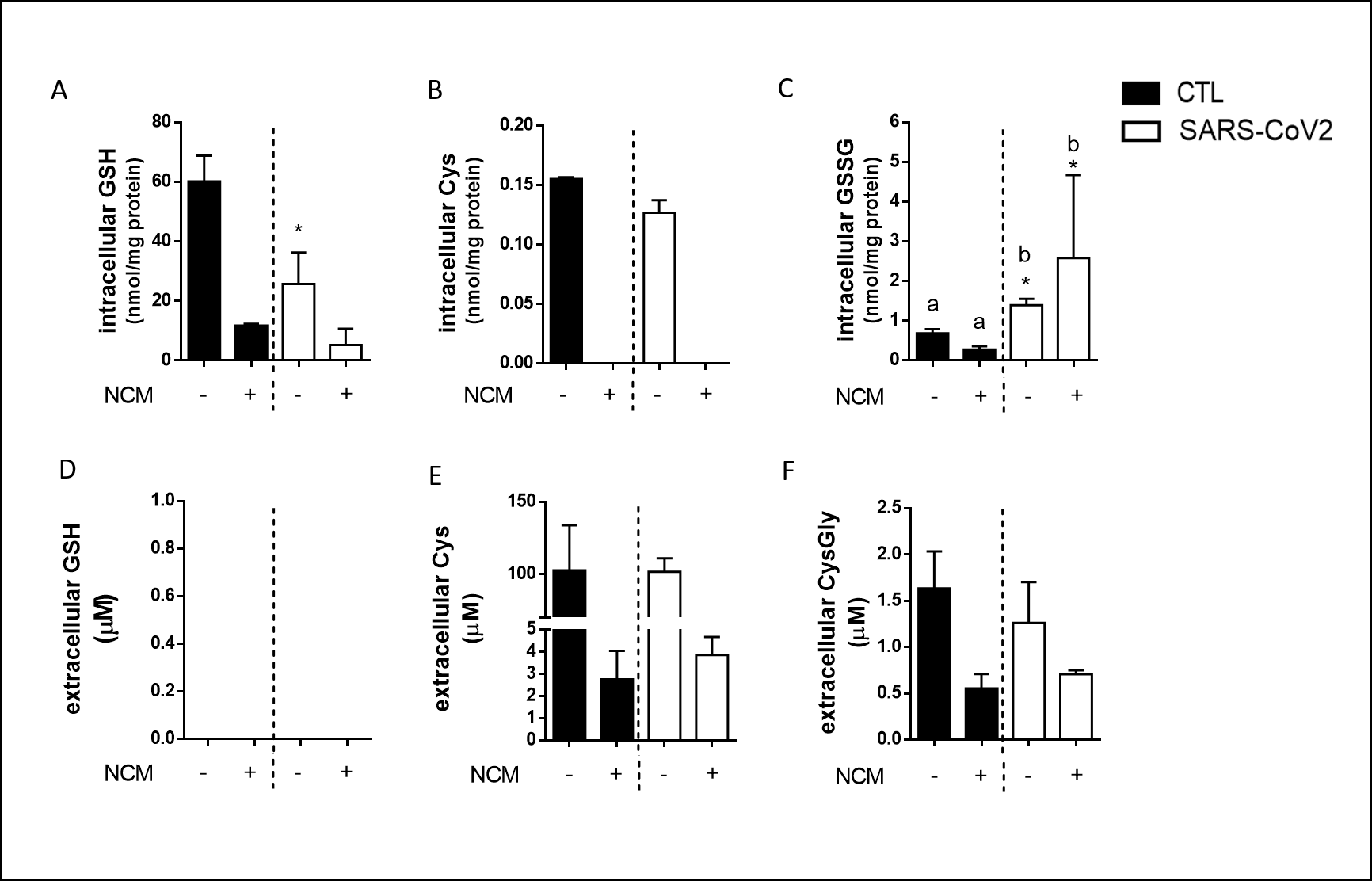


**Supplementary Figure 2. Cellular and extracellular thiols in VERO-E6 cells exposed to SARS-CoV2 in complete or non-complete medium**. The data in this chart provides a direct comparison between the response of cellular and extracellular thiols to SARS-CoV2 infection in cells maintained for 24 hrs in culture in the presence or absence of sulphur-containing amino acids. The absence of these amino acids (which is identified with the term “non-complete medium” or NCM) has been adopted in Figure 2 (see the scheme of Figure 2A) to allow reliable measurements of cellular thiol efflux avoiding the interference of Cys that is present at high micromolar levels in standard culture media [22]. The data include: **A)** Intracellular GSH; **B)** intracellular Cys; **C)** intracellular GSSG; **D)** extracellular GSH; **E)** extracellular Cys; **F)** extracellular CysGly. Control test (complete medium) *vs* NCM: **a** (control test with uninfected cells, black bars) and **b** (SARS-CoV-2 infected cells, white bars) = p<0.05; (*) control cells (uninfected cells, black bars) *vs* SARS-CoV2 infected cells (white bars) in the same medium = p<0.05.


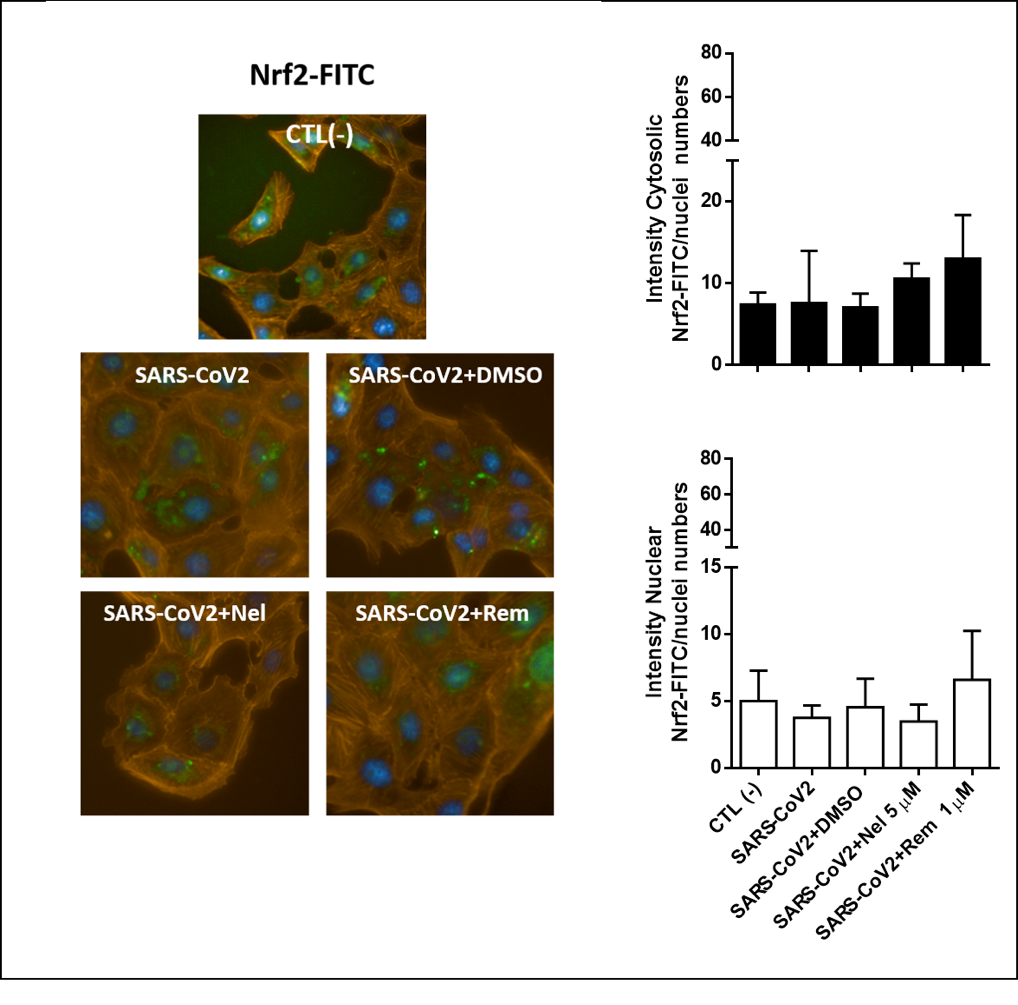


**Supplementary Figure 3. Nrf2 levels in SARS-CoV2 infected VERO-E6 cells 24hpi treated with Nelfinavir (Nel) or Remdesivir (Rem).** Nrf2 semi-quantitative fluorescence analysis 24 hpi. Fluorophores were FITC (green) for Nrf2 protein labelling, DAPI (blue) for nuclei and Phalloidin-Alexa Fluor595 (orange) for the cytosolic space.


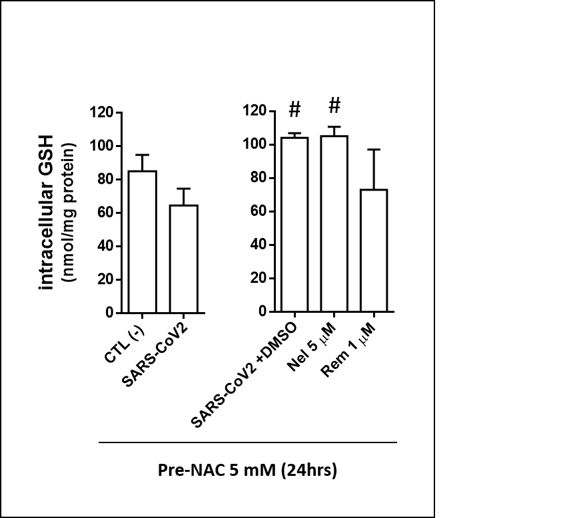


**Supplementary Figure 4. Cellular levels of GSH in VERO-E6 cells pre-treated with NAC and exposed to SARS-CoV-2 infection.** Pre-treatment with NAC (5 mM) was carried out for 24hrs and then the cells were infected with SARS-CoV-2 (MOI:0.0035) in complete medium in the presence or absence of antiviral drugs. Cellular GSH was measured 24 hpi. (#) SARS-CoV2 *vs* SARS-CoV2 ± vehicle (DMSO) or drug treatments = p<0.05.


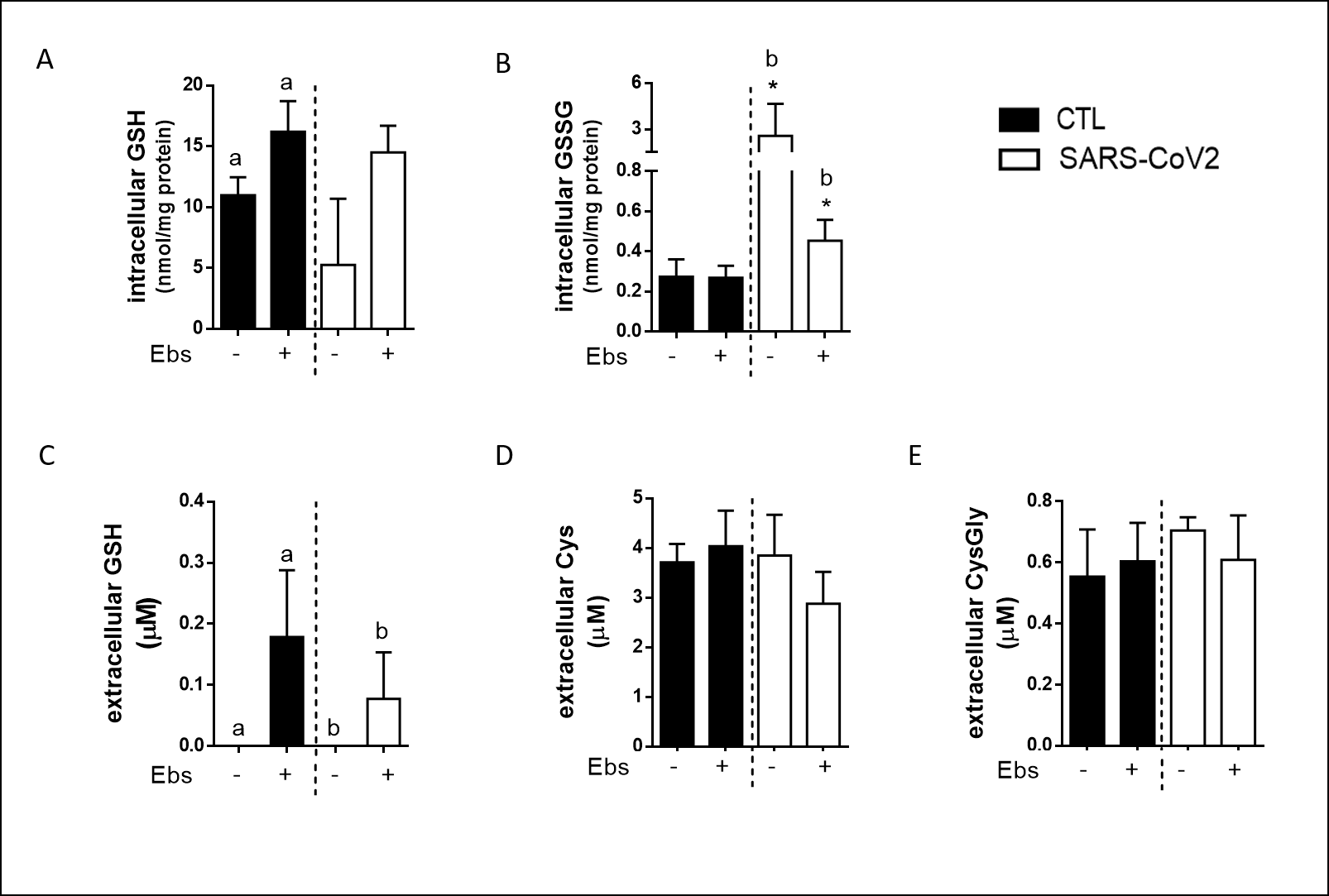


**Supplementary Figure 5. Effect of Ebselen on cellular thiols of SARS-CoV2 infected VERO-E6 cells.**

VERO E6 cells were infected with SARS-CoV-2 as described in Figure 1 and maintained for 24 hrs in medium deprived of sulphur-containing amino acids (non-complete medium or NCM) supplemented with 25 µM Ebselen (Ebs). **A)** Intracellular GSH; **B)** intracellular GSSG; **C)** extracellular GSH, **D)** extracellular Cys; **E)** extracellular CysGly. NCM *vs* Ebs: **a** (control test with uninfected cells, black bars) and **b** (SARS-CoV2 infected cells, white bars) = p<0.05; (*) control cells (uninfected cells, black bars) *vs* SARS-CoV2 infected cells (white bars) in the same treatment conditions = p<0.05.

**Suppl. Figure 6. Schematic representation of the alterations that SARS-CoV-2 infection generates in the metabolism and redox function of cellular glutathione.**

**Supplementary Tables**

**Suppl. Table 1. Mean levels of intracellular and extracellular thiols in SARS-CoV2 infected VERO-E6 cells.**

|  | **VERO E6 cells**  **(24 hrs)** | **mean levels of intracellular thiols (nmol/mg protein)** | **mean levels of extracellular thiols**  **(µM)** |
| --- | --- | --- | --- |
| Complete medium  (CM) | **CTL** | 60.3 | 106.7 |
|  | **SARS-CoV2** | 25.8 | 11.2 |
| Non-complete medium (NCM) | **CTL** | 11.0 | 4.0 |
|  | **SARS-CoV2** | 5.3 | 4.9 |
| Co-NAC  NCM | **CTL** | 3.5 | 178.3 |
|  | **SARS-CoV2** | 2.0 | 194.1 |
| Pre-NAC  24hrs+ NCM | **CTL** | 62.4 | 6.7 |
|  | **SARS-CoV2** | 31.8 | 3.9 |
| Mean levels of thiols presented in this chart represent the sum of all the thiol species measured in the experiment shown in Figure 1 and 2, and include data obtained in NAC-treated cells (treatments were performed according to the co-NAC and pre-NAC protocols described in the scheme of Figure 2A). | | | |

**Suppl. Table 2.** I**ntracellular levels of the oxidized forms of Cys (Cyss) and γ-GluCys (γ-GluCyss) in VERO-E6 cells infected with SARS-CoV2.**

| **VERO E6 cells**  **(24 hpi)** | **Cyss**  **(nmol/mg of protein)** | **γ-GluCyss**  **(nmol/mg of protein)** |
| --- | --- | --- |
| **CTL** | 0.67 ± 0.13 | 0.13 ± 0.05 |
| **SARS-CoV2** | 1.03 ± 0.09 | 0.22 ± 0.08 |
| Data are expressed as mean ± SD of five independent experiments. | | |

**Suppl. Table 3. CC_50_ of antiviral drugs.**

| **Antiviral drug** | **CC_50_, µM** |
| --- | --- |
| Nelfinavir (Nel) | 46.9 ± 5.2 |
| Remdesivir (Rem) | >100 |
| Saquinavir mesylate (SM) | 54.8 ± 8.1 |
| Indinavir sulfate (IS) | >100 |
| Cell viability was measured by MTT assay after 24 hrs post-treatment. The experiments were performed in triplicates. | |
